# Identification and Characterization of miRNA Transcriptome in Potato by High-Throughput Sequencing

**DOI:** 10.1371/journal.pone.0057233

**Published:** 2013-02-21

**Authors:** Runxuan Zhang, David Marshall, Glenn J. Bryan, Csaba Hornyik

**Affiliations:** The James Hutton Institute, Invergowrie, Dundee, United Kingdom; University of Nottingham, United Kingdom

## Abstract

Micro RNAs (miRNAs) represent a class of short, non-coding, endogenous RNAs which play important roles in post-transcriptional regulation of gene expression. While the diverse functions of miRNAs in model plants have been well studied, the impact of miRNAs in crop plant biology is poorly understood. Here we used high-throughput sequencing and bioinformatics analysis to analyze miRNAs in the tuber bearing crop potato (*Solanum tuberosum*). Small RNAs were analysed from leaf and stolon tissues. 28 conserved miRNA families were found and potato-specific miRNAs were identified and validated by RNA gel blot hybridization. The size, origin and predicted targets of conserved and potato specific miRNAs are described. The large number of miRNAs and complex population of small RNAs in potato suggest important roles for these non-coding RNAs in diverse physiological and metabolic pathways.

## Introduction

The recent discovery of small RNAs and their widespread roles in post-transcriptional gene regulation has changed our basic understanding of how genes are regulated during development and in different biological processes. These RNAs represent an additional layer in regulation of gene expression. The pathways and key enzyme components are well described and are highly conserved in plants and animals [1]. Micro RNAs (miRNAs) comprise one class of the diverse small RNA families. miRNAs are short (21–24nt) in length, single stranded, non-coding RNAs produced from an RNA Polymerase II transcript (pri-miRNA) with a strong secondary structure (stem-loop) [2], [3]. In plants Dicer-like 1 (DCL1) protein (RNase III endoribonuclease) cleaves this secondary structure and produces a hairpin RNA molecule (pre-miRNA) for further cleavage by DCL1 resulting in a double stranded intermediate RNA. One strand of this RNA is incorporated into the effector complex (RISC - RNA Induced Silencing Complex), this is the mature miRNA (guide strand); the other strand is known as the ‘star’ sequence or passenger strand (miRNA*) and is usually degraded but sometimes also functions as a miRNA. The core component of RISC is one of the Argonaute proteins which forms a complex with the miRNA; which guides the effector complex to the target RNA directing RNA degradation or translational inhibition [4].

Increasing evidence shows that miRNAs play important roles in developmental processes and gene regulation upon biotic and abiotic stresses [5], [6], [7], [8]. miRNAs were investigated in detail in model plants [9], [10], [11] but with the development of ‘second generation’ sequencing techniques it has become feasible to carry out small RNA studies in crop species. Many important crops have been studied and both species-specific and conserved miRNAs have been discovered in, for example, rice, tomato, cucumber, grapevine, oilseed rape and maize allowing detailed biological studies on gene regulation [12], [13], [14], [15], [16], [17], [18]. The availability of genomic sequence supports genome-wide small RNA studies providing a global view of small RNA function in different species.

Potato is an important global food crop which is cultivated for its underground storage stems (tubers), rich in starch and nutrients and is unique among the major crops in tuber formation. The importance of potato globally is shown by the ∼330 million tons production in 2010 (http://www.fao.org). Moreover, potato is being grown more extensively in developing countries with rapidly growing populations, such as those in South East Asia, so it is playing an increasing role in addressing food security issues. It is extremely important to understand the as yet poorly known molecular events in tuber development, which ultimately impact on the breeding of cultivars with improved tuber characteristics (yield, size distribution, shape etc.). There is a great deal of current interest and research in the molecular signals which promote tuberization in potato [19], [20]. The availability of the potato genome sequence allows the possibility for genome-wide studies [21]. Some potato miRNAs were described in previous studies, but these were mainly predicted using previously available sequence data [22], [23], [24], [25]. Other studies, using large scale analysis focused only on finding conserved miRNAs in potato [26]. Few miRNAs were studied in depth as having a role in tuberization or regulation of defence genes in potato [27], [28].Taking advantage of the recently published potato genome we have used Illumina sequencing technology and existing and newly developed bioinformatics tools to identify miRNAs from potato and predict their targets. We characterized small RNAs from leaf and stolon tissues in potato; confirmed 28 conserved miRNA families and found 120 potato-specific miRNA families. Potato-specific miRNAs were validated and target RNAs were predicted for the majority of predicted miRNAs.

## Results

### Analysis of potato small RNAs

**Figure 1:**
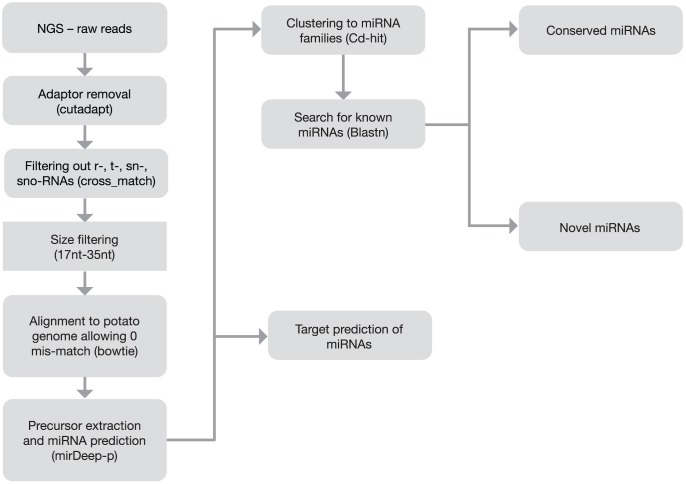
Flowchart for the prediction of miRNAs and their target RNAs in potato.

*Solanum tuberosum* group *Andigena* (clone ADG573) was used for small RNA library preparation and next generation sequencing. This clone was developed at The James Hutton Institute from the Commonwealth Potato Collection (CPC, http://germinate.scri.ac.uk/germinate_cpc/app/index.pl). ADG573 has the feature of being able to tuberize under short days (SD) but not under long days (LD), which is typical of germplasm originating from the Andes in South America. From 12 libraries of leaf and stolon materials using LD and SD conditions with three biological replicates (see Material and Methods) about 60 million raw reads were obtained and further analysed. Firstly reads were quality filtered, adaptors were removed and ribosomal RNAs (rRNAs), transfer RNAs (tRNAs), small nuclear RNAs (snRNAs) and small nucleolar RNAs (snoRNAs) were filtered out from the database (Fig. 1, Table 1, for details see Material and Methods).

**Table 1:** Summary of reads and unique sequences in small RNA libraries.

	# reads	# unique sequences
Raw reads	59,829,936	
Adaptor removed	59,413,448	8,475,945
r-, t-, sn-, sno-RNA removed	35,991,259	7,192,667
17nt-35nt filtering	18,940,842	6,143,665

**Figure 2:**
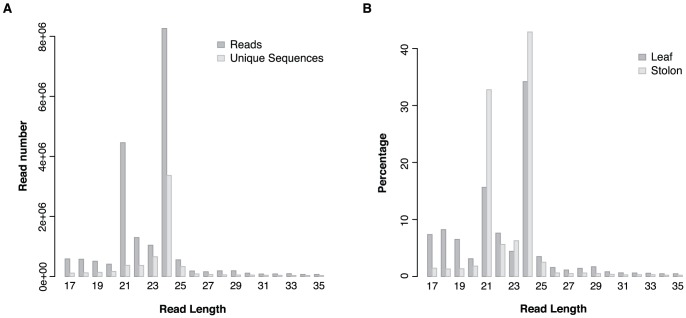
Size distribution of small RNA sequences. (A) Reads and unique sequence distribution in all the small RNA libraries. 24 and 21nt length reads are the most abundant reads among the small RNAs. (B) Size distribution of reads originating from leaf (dark grey) and stolon (light grey) tissues expressed as a percentage of total reads. 24 and 21nt length reads are dominant in both of the tissues; in stolon tissues there are more reads for 21, 23 and 24nt length.

In order to characterize potato small RNAs, the size distribution of reads was analysed and these data are summarized in Figure 2A. The majority of reads are in the range of 21–24 nt in length, with 24 nt being the most abundant group of small RNAs. Read number and unique read number are the highest in this group but for the size of 21 nt which is characteristic for canonical miRNAs we found a high ratio for reads/unique reads reflecting the abundance of these regulatory RNAs in plants. The dominance of the 24 nt read length is consistent with previous reports from other species such as *Arabidopsis thaliana*, *Medicago truncatula*, *Oryza sativa*, tomato, cucumber, maize and *Populus trichocarpa* [13], [15], [18], [29], [30], [31], [32]. 24nt long small RNAs exhibited high sequence diversity consistent with the origin of this size class (mainly repeat associated siRNAs)[33], but we found the highest diversity for the 23nt small RNAs compared to total read number (Fig. 2A). A similar small RNA read distribution was found in different tissues when reads from leaf and stolon derived libraries were compared (Fig. 2B). Small RNA reads are more evenly distributed between 17–35nt in leaf tissues. However, stolon samples showed sharper peaks at the size of 21, 23 and 24 nts.

The sequenced ‘DM’ genome assembly was used to align the reads to the reference sequence and no mismatches were allowed in the analysis using Bowtie software [34]. Reads were visualized by Tablet software on the potato super-scaffolds to better understand the distribution of small RNAs in their genomic context [21], [35].

### Identification of conserved and non-conserved miRNAs in potato

**Figure 3:**
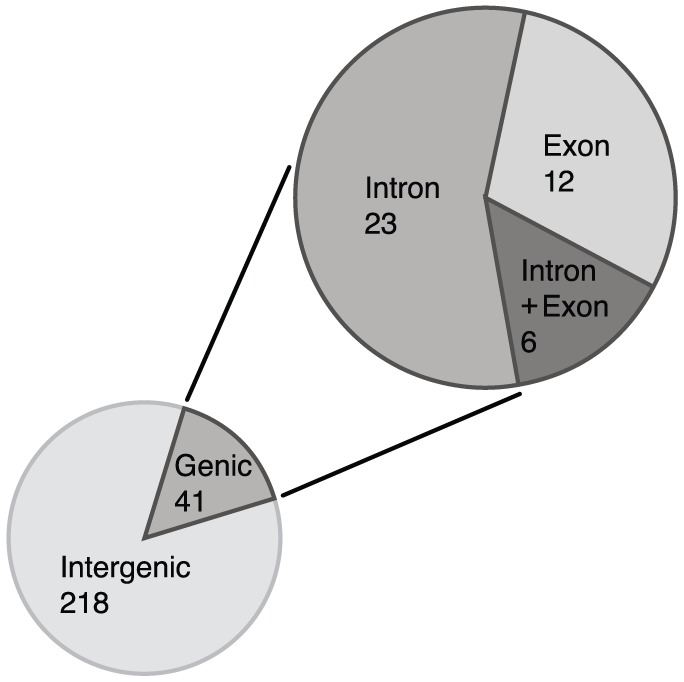
Origin of miRNA precursors. The majority of miRNA precursors are transcribed from intergenic regions. miRNA precursors originating from coding regions locates mainly in introns.

In order to identify conserved and potato specific miRNAs in the database we analysed the data obtained from high-throughput small RNA sequencing. We applied miRDeep-P and predicted miRNAs candidates [36]. First, 250 nt windows around of the aligned reads were excised from the genome and the secondary structures of the potential precursors were predicted using RNAfold [37]. Predicted miRNAs were retrieved using mirDeep-P and redundant predicted miRNAs were removed using an updated set of criteria for plant miRNAs [38]: 1) the miRNA and miRNA* are derived from opposite stem-arms and they form a duplex with two nucleotide 3′ overhang; 2) base-pairing between the miRNA and miRNA* has no more than 4 mismatches; 3) there is no more than one asymmetric bulge within the miRNA and miRNA* duplex and the bulge is no more than 2 nucleotides in size. All the predicted miRNA sequences with less than 5 reads in all libraries were also removed ending up with 259 predicted plant miRNAs in our database (Table S1). The read number of predicted miRNAs shows high diversity, ranging between 8–741,106 and we found reads that fall in the star sequence regions in the dataset for 66% of predicted miRNAs (Table S1). Mapping of reads to the potato genome annotation suggests that 84% of the pre-miRNAs locate in intergenic regions (Fig. 3), suggesting that a high proportion of miRNAs are encoded by non-annotated genes or noncoding RNAs. The majority of pre-miRNAs which are encoded in genic regions locate in introns and only 12 out of 41 are in exon sequences. Six miRNA precursors locate both in intron and exon sequences.

**Figure 4:**
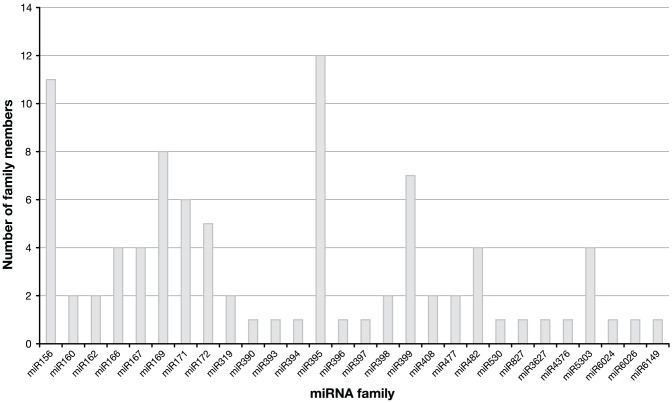
Distribution of conserved miRNA family members in potato. Graphical representation of the different members of conserved miRNA families found in potato by sequencing and bioinformatics prediction.

To identify the potential miRNA families, we have used Cd-hit to cluster the predicted potato miRNA candidates [39]. Altogether, 159 miRNA families have been identified. Using Blastn we identified 39 potato miRNA families which were conserved in other plant species allowing only three mismatches or gaps including overhanging nucleotides at the 5′ or 3′ end between the predicted potato and the known miRNAs. The clusters with homologues that fall into the same family were merged giving a final number of 28 conserved miRNA families in potato discovered in this study (Fig. 4, Table S2.). This analysis shows that similar numbers of miRNA family members were found in potato for the conserved miRNA families compared to other species. The smallest read number (72) was found for miR827 but miR319, miR396, and miR166 show high abundance (>11,000 each, data not shown). Our analysis indicated 120 miRNA families which are not conserved among species using our filtering criteria. The majority (80%) have only one member and the largest family has 8 members (Fig. S1, Table S2).

**Figure 5:**
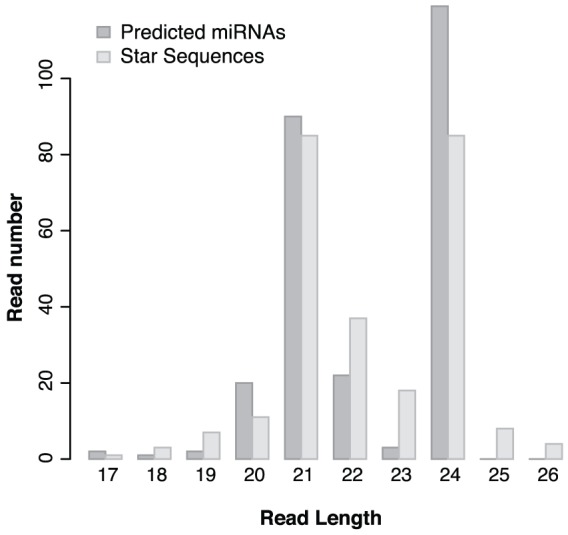
Reads length analysis of the predicted unique miRNA and star (miRNA*) sequences. Graph shows that most miRNAs and star sequences have 24 and 21 nt length. Star sequences show different distribution compared to miRNAs; there are less star sequences with 17, 20, 21 and 24 nt length but more in other length categories.

We have analysed the size distribution of predicted unique miRNAs and their star sequences (Fig. 5). The 24 nt length category has the highest number of miRNAs and 21 nt long miRNAs are represented in our samples with high frequency as well. According to the size distribution the most abundant star sequence read length categories are 24 and 21 nt similar to the predicted mature miRNAs. However, with 22 and 23 nt length we found more star sequences than miRNAs and in other examined length categories (18–19 and 25–26 nt) the star sequences are also more numerous than miRNAs.

### Validation of potato specific miRNAs

**Figure 6:**
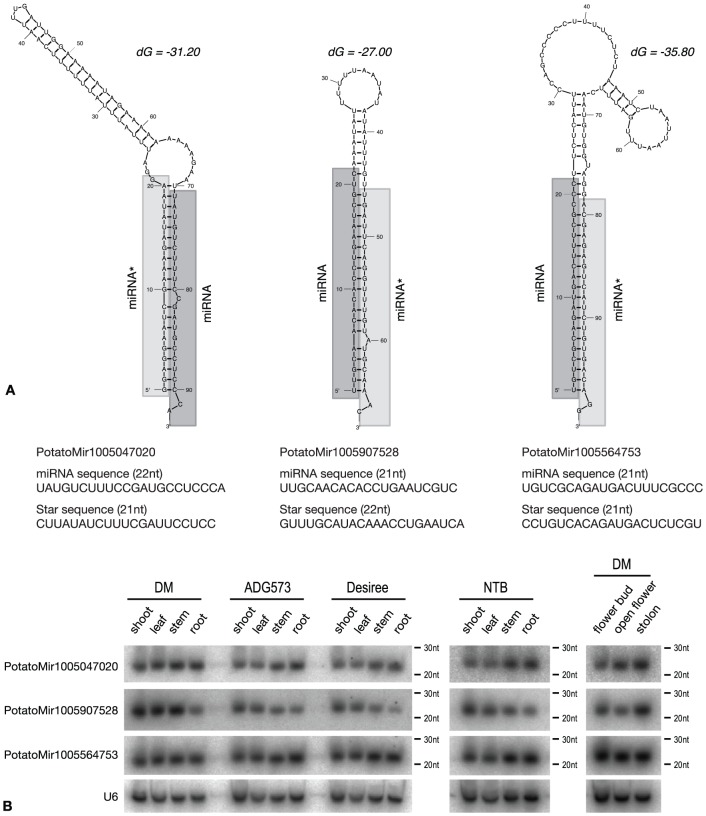
Bioinformatics prediction and RNA gel blot detection of potato specific miRNAs. (A) Secondary structure of predicted pre-miRNAs (Mfold) showing the length and sequence details below the diagrams. miRNA sequences are highlighted with dark grey, star sequences with light grey colours. (B) RNA gel blot hybridization of radioactively labelled probes against novel potato specific miRNAs in different species and tissues.

In order to validate the effectiveness of our bioinformatics pipeline we chose three predicted miRNAs with no matches in other species (PotatoMir1005047020, PotatoMir1005564753, PotatoMir1005907528). These miRNAs have relatively high read numbers in our database (4997, 3830 and 3771 respectively) and each originates from one locus in the genome. Figure 6A shows the predicted secondary structure of pre-miRNAs with the minimum free energy ranging from −27.00 to −35.80 dG. All of the examined pre-miRNAs could be folded into a hairpin structure with extensive base pairing in the stem. The length of miRNA and star sequence is different in the cases of two miRNAs which is indicated by the bulge in the stem region.

To validate the presence of these miRNAs in plant tissues RNA gel blot hybridization was performed. All the examined miRNAs are highly expressed in the examined tissues (shoot, leaf, stem, root, flower bud, open flower and stolon)(Fig. 6B). We have used not only *S. tuberosum* group *Andigena* plants but also DM (doubled monoploid) which was used for the potato genome sequencing project [21], Désirée a commonly used cultivar and NTB369 (*S. tuberosum* group *Andigena* ‘Neotuberosum’ clone selected for long day tuberization). PotatoMir1005047020 showed similar expression pattern in different tissues in all the plants but PotatoMir1005907528 had lower levels in roots and higher in stolons. PotatoMir1005564753 had strong expression in every tissue in the different plants. These analyses strongly suggest that the examined miRNAs exist in plants and have high expression levels in several potato tissues.

### Target prediction for conserved and potato specific miRNA

With the aim to better understand the biological role of conserved and potato specific miRNAs we searched for putative target genes by using a plant small RNA target analysis tool: psRNATarget [40] with the transcript sequences of the potato genome as a reference set. Table S3 shows the predicted transcripts for each miRNA and other information originating from the prediction: unpaired energy, aligned miRNA and transcript sequence, annotated gene function and the inhibition type: cleavage or translational inhibition. Target transcripts were predicted for 241 out of 259 miRNAs (93.1%). Only 18 miRNAs have no targets according to our analysis, but this may be due to use of a relatively stringent cut-off for the predictions to reduce number of false positive hits. For the majority of miRNAs more than one transcript was identified as a possible target. When more relaxed filtering conditions were applied for target prediction we found more potential targets for the miRNAs (Table S3).

To prove the power of our prediction we searched among the predicted targets of miR172 for the known target *RAP1* (*Relative to APETALA2 1*) [27]. Transcript PGSC0003DMT400071732 (corresponds to *RAP1*) was found for all the miR172 family members as a predicted target with low expectation score and unpaired energy suggesting it to be a highly likely target. Additionally, the NL25 protein disease resistance gene transcript (PGSC0003DMT400006234) is targeted by miR482b which is present in our prediction [28]. Further analysing the validated potato specific miRNAs we found targets for two of them: our prediction shows that PotatoMir1005907528 may target mRNAs encoding Galactose oxidase and F-box domain containing proteins and one transcript originating from a gene with unknown function. PotatoMir1005564753 may target transcripts mainly originating from genes with unknown function and Aldehyde dehydrogenase or Exostosin transcripts (Table S4) suggesting a role for these miRNAs in different biochemical pathways. The miRNA-target transcript dataset which we have generated contains the majority of predicted miRNAs with possible targets in potato and gives a unique possibility to find miRNA-target interaction in essential biological processes (Table S3).

## Discussion

miRNAs play key roles in most biological processes in animals and plants [41], [42]. Many of the new and evolutionary conserved miRNAs were detected in different species especially using the high-throughput sequencing technologies but most of the species-specific miRNAs that have low expression levels, are expressed in different tissues or have spatio-temporal expression remain, as yet, unidentified [1]. By taking advantage of the recently sequenced potato genome the aim of this study was to predict miRNAs and their targets in this important crop. We have used a high-throughput approach to identify the small RNA transcriptome of potato and identify miRNAs using these data. For target prediction the newly annotated potato genome transcript data was used [21] enabling a view of the high number of transcripts which might be targeted by miRNAs.

The small RNA analysis of potato showed that the 24 nt length class dominates in the dataset in total number and unique sequences strongly suggesting that small RNA directed heterochromatin silencing plays an important role in this crop similar to other species [13], [29]. The high read number of the 21 nt length class with low number of unique reads suggests an important role for this size class where many miRNAs could be found. The search for miRNAs in potato revealed 259 distinct miRNAs. The majority appear to mature from transcripts of intergenic regions or from non-annotated genes of the potato genome which is in line with a previous finding that most plant miRNAs are intergenic [42]. Our analysis shows that the majority of pre-miRNAs originating from coding sequences are encoded in introns. We have used relatively strict rules to cluster the predicted miRNAs into families and identify conserved miRNAs among them (maximum of 3 mismatches and gaps were allowed including overhanging nucleotides at the 5′ or 3′end) [38]. This might be one reason why only 28 conserved families and a relatively high number of potato specific miRNA families have been identified. A second important reason could be that the current study was limited to only leaf and stolon tissues so many other miRNAs which show different spatio-temporal expression in potato might be undetected.

Most predicted miRNAs are of 21 and 24 nt in length. 21 nt miRNAs have the canonical size for small RNAs derived from DCL1 processing but a little variation in size might originate from the inaccuracy of DCL1 processing [9] which we could see clearly in the reads aligned to the genome. The importance of DCL3 and other DCLs in processing of miRNAs of potato could be reflected in the high abundance of 24 nt length miRNAs because DCL3 can generate longer miRNAs (23–25 nt) than usual [43]. This longer size class might represent a group of miRNAs which originate from recently evolved MIR genes. In contrast, the star sequences show lower abundance at 21 and 24 nt but more star sequences were found with 22 and 23nt length than miRNAs (Fig. 5). Also, we found more star sequences in length of 18–19 and 25–26nt. These differences in size and the resulting ‘bulge’ in the hairpin might suggest that a large number of miRNAs has the potential to initiate secondary siRNA generation [44], [45], [46], [47].

The prediction of miRNAs in our study, in contrast with previous reports, is based on results from high-throughput small RNA sequencing. Zhang et al. (2009) predicted 21 miRNA families but our study predicted 14 additional conserved miRNA families in potato. For 6 families we have predicted more members in the same family but our high-throughput study could not confirm the prediction for 9 of the reported miRNA families. Similarly, twelve miRNA families were found in common with another study [24]. We predict 16 additional conserved miRNA families, however, our analysis does not support the prediction of 36 more miRNA families by these authors. In comparison with the study of Kim et al. (2010), we found six common families, as well as an additional twenty-two in our study, although we could not confirm their prediction for 14 miRNA families. Xie et al. (2010) confirmed the expression of 12 different conserved miRNAs by qRT-PCR and we have found 11 of these by high-throughput sequencing. Our analysis predicts 10 more conserved miRNA families in potato (and more members for the identified families). The prediction of 60 miRNA families in the same study is not supported by our prediction based on sequencing results. The authors of published studies have not analysed miRNAs with a length of 24nt, this category being strongly supported by our sequencing data. The difference in number of conserved miRNA families between our and the previous studies could be due to the different methods we used for bioinformatics analysis and the available sequence data. Additionally, different types of samples might have been used and collected at different time points. Similarly, the growing conditions could also strongly influence the presence and abundance of miRNAs. The strength of our prediction is demonstrated compared to previous studies; using the potato genome sequence we predicted more members for the conserved miRNA families and potato specific miRNAs were predicted, which has not been previously shown. We have predicted more target RNAs for the miRNAs giving a better view about the potential role of miRNA directed gene regulation than in previous studies [22], [23], [24], [25].

Potential targets with a wide variety of predicted functions were identified for the miRNAs in potato: transcription factors, genes with a role in defence mechanisms, kinases and ion homeostasis genes. We have predicted the mRNA/gene targets of many potato miRNAs. Some have known functions in flowering and tuberization (miR172) or guiding cleavage of transcripts of immune receptors (miR482) [27], [28], [48]. We have predicted miRNAs for many well described miRNA families from other species (Fig. 4., Table S3). Additionally, potato specific miRNA targets were predicted in this study. Further studies focusing on miRNA directed gene regulation and finding target genes in diverse biological pathways are necessary to elucidate the functional importance of predicted miRNA-target RNA relations.

In summary, we have identified conserved and potato specific miRNAs and their targets for the first time at a genome-wide level. Using high-throughput sequencing technology and taking advantage of the recently published potato genome [21] a pipeline was adopted and developed to analyse small RNA data from potato which could be helpful for other studies in potato or different *Solanaceae* species.

## Materials and Methods

### Plant material

*Solanum tuberosum* group *Andigena* (line ADG573) was used for the experiments. Plants were propagated *in vitro* by single-node cuttings on MS 20 [49]. Plantlets were planted into compost and grown for four weeks in a glasshouse under LD (16 h light/8 h dark) conditions. Plants were transferred into growth cabinets for LD (16 h light/8 h dark) and SD (8 h light/16 dark) at 20 °C with light intensity of 170 µmol m^−2^ sec^−1^. After the shift to cabinets, leaf (8 days at SD and LD) and stolon (12 and 16 days of SD plants) materials were collected 4–6 hours after dawn. For miRNA validation DM (doubled monoploid, *S. tuberosum* group *Phureja* DM1-3 516 R44) [21], ADG573, NTB369 (*Solanum tuberosum* group *Andigena*, Neotuberosum) and Désirée were used. Plants were grown in glasshouse under LD conditions for four weeks after *in vitro* propagation.

### RNA analysis

Total RNA was extracted from leaf and stolon material using Plant/Fungi Total RNA Purification Kit (Norgen, Canada) according to the manufacturer's instructions. An Illumina TruSeq Small RNA sample prep kit (Illumina, USA) was used for the preparation of small RNA libraries according to the manufacturer's instructions starting with 1.5 µg total RNA and Illumina GAIIX sequencer for sequencing.

For RNA gel blot hybridization of miRNAs total RNA (10 µg for leaf and shoot tissues, 5 µg for flower, stem, root and stolon tissues) was separated by 15% polyacrylamide (19:1) gel with 8M urea and 1 × MOPS (20 mM MOPS/NaOH, pH7) buffer. RNA markers (Decade RNA markers, Ambion, USA) were end labelled by ^32^γ-ATP. RNA was blotted onto Hybond-N membrane (Amersham, GE Healthcare, UK) using a Panther™ Semi-dry Electroblotter, HEP-1 (Thermo Scientific Owl Separation Systems, USA) and cross-linked by N-(3-Dimethylaminopropyl)-N′-ethylcarbodiimide hydrochloride (EDC, Sigma-Aldrich, USA) [50]. DNA oligos (20 pmol) were end-labelled by ^32^γ-ATP using T4 polynucleotide kinase (NEB, USA) to visualize miRNAs. For sequences see Table S5. Hybridization was performed in 50% formamide, 5XSSPE (1XSSPE - 0.115M NaCl, 10 mM sodium phosphate, and 1 mM EDTA, pH7.4), 5XDenhardt's solution (1XDenhardt's - 0.02% Ficoll, 0.02% polyvinylpyrrolidone, and 0.02% BSA), and 0.5% SDS with competitor Herring sperm DNA (0.1 mg/ml, Sigma-Aldrich, USA). After overnight incubation at 37 °C, the membrane was washed twice in 2XSSC and 0.1% SDS for 15 min at 37 °C. After hybridization signals were detected by phosphorimager plate visualized by FLA-7000 Fluorescent Image Analyzing System (Fujifilm, USA). Secondary pre-miRNA structure of validated miRNAs were predicted by Mfold and the lowest free energy form is shown in Figure 6A [51].

### Bioinformatics analysis of small RNAs

Quality trimming and adaptor removal of the Illumina reads were carried out using Cutadapt (http://code.google.com/p/cutadapt/). First, low-quality ends were trimmed by applying a quality score threshold of 20. In the applied trimming method, the threshold is subtracted from all quality scores and partial sums from all indices to the end of the sequence are then computed. Sequences are cut at the indices where the sums are minimal. Adaptors were then removed by matching the first (for 3′ adaptors) and last (for 5′ adaptors) 8 nt of the adaptor sequences while allowing 1 nt of mismatch maximum.

Sequences were then searched against ribosomal, transfer RNAs, snRNAs and snoRNAs from Rfam, the Arabidopsis tRNA databases and plant rRNA and tRNA sequences from EMBL using cross_match (http://www.phrap.org/phredphrapconsed.html). The sequences aligned to the above databases with at least 10 matched nts and a minimum alignment score of 10 were excluded from further analysis. Sequences, shorter than 17nt and longer than 35 were also discarded.

#### Identification of candidate miRNAs

The resulting 18,940,842 reads were firstly processed into 6,143,665 non-redundant sequences with read counts associated with each sequence. 3,280,908 unique sequences were successfully aligned to the potato superscaffolds (http://potatogenomics.plantbiology.msu.edu/index.html) on all of the plausible positions using Bowtie with perfect matches (0 mismatches). Then the sequences that were mapped to multiple positions in the genome were filtered based on the maximum size of potato miRNA families. The threshold was set at 30 to recover small RNAs with relatively large family sizes. 4,006,195 flanking regions of the 3,113,450 filtered sequences were retrieved from the genome using a window of 250 bps. Then the secondary structures of the flanking sequences were predicted using RNAfold (http://www.tbi.univie.ac.at/RNA/man/RNAfold.html, [52]). 25,457,070 signatures, which are numbers and relative positions of reads in potenitial miRNA precursors [53], were generated by aligning the sequences to the flanking sequences and then 1102 predicted miRNAs were retrieved using mirDeep-P by applying Score> = 1. The redundant predicted miRNAs were then removed and updated criteria [38] for plant miRNAs were employed to filter the results: 1) the miRNA and miRNA* are derived from opposite stem-arms and they form a duplex with two nucleotide 3′ overhang; 2) base-pairing between the miRNA and miRNA* has no more than 4 mismatches; 3) there is no more than one asymmetric bulge within the miRNA and miRNA* duplex and the bulge is no more than 2 nucleotides in size.Finally, all the predicted miRNA sequences with less than 5 reads were removed, ending up with 259 predicted plant miRNAs. Above analysis was implemented using tools from mirDeep-P, which was developed by modifying miRDeep [53] with a plant-specific scoring system and filtering criteria [36].

#### Identification of miRNAs families in Potato

To identify the potential miRNA families in potato, we have used Cd-hit to cluster the predicted potato miRNA candidates. A word length of 7 and a sequence identity threshold of 0.9 were selected to cluster the predicted miRNA sequences. Altogether, 159 families have been found in the 259 predicted potato miRNA candidates.

#### Conserved miRNAs in Potato

To identify conserved miRNAs in potato, Blastn was used to match the predicted potato miRNA candidates to all the 5940 plant mature miRNAs from mirBase Release 19 [54]. In our analysis, word size was set as 7 for the blastn search. The maximum for the sum of mismatches and gaps were set at 3 to filter the alignment results. When blast search indicated more than one miRNA family hits the one was selected which showed least mismatches. 39 potato miRNA families were conserved in other plant species. The clusters with homologues that fall into the same family were merged and finally 28 conserved miRNA families in potato were confirmed.

#### miRNA target predictions

A plant small RNA target analysis tool psRNATarget [40] was employed to predict the targets of the putative 259 miRNAs in the nucleotide sequences of all transcript sequences from the Potato Genome Consortium (PGSC_DM_v3.4_transcript-update.fasta). psRNATarget predicts small RNA targets by reverse complementary matching between small RNA and target transcripts and evaluating the target site accessibility by calculating unpaired energy required to open secondary structure around the small RNA's target site [40]. It also reports the translational inhibition or cleavage degradation by presence/absence of a mismatch in the central complementary region of the small RNA sequence. To control the false positive rates, a conservative threshold for maximum expectation value was set at 2.0 and hsp size (length for complementary scoring) at 17. Target accessibility was set at 25. 17 bp upstream and 13 bp downstream of the target site were used for target accessibility analysis. The range of central mismatch leading to translation inhibition is between 9 and 11 nt.

We also carried out the same target prediction analysis using psRNATarget allowing less stringent pairing between miRNA and its target transcripts. The maximum expectation score measures the complementarity between small RNA sequences and its target transcripts. Setting the maximum expection score to 3.0 allows more miRNA/target pairs to be identified but possibly with higher false discovery rates. The result for such prediction is shown in Table S3 to cater for studies where discovery of more potential miRNA/target is of interest.

Accession number of short RNA dataset: GEO accession GSE39230.

## Supporting Information

Figure S1 **Distribution of potato specific miRNA families by numbers of members.** Graph legend indicates the number of members in different families. The majority of newly identified potato specific miRNA families have one member.
(EPS)

Table S1 **Predicted known and new miRNAs in potato.** The table shows the predicted miRNAs according to the family number (family #); the super-scaffold location (chromosome id); the direction of chromosome strand where the miRNA maps (strand direction); read and precursor identification number according to the analysis (reads id, precursor id); the homologue of miRNA (miRNA family); read number for miRNAs and star sequences (read #, star read #); the location of miRNA and precursor RNA on the super-scaffold (mature miRNA location, precursor location) and the mature, precursor and star sequences. Total read numbers of each predicted miRNAs are indicated for the different tissues which were analysed. During the analysis DNA equivalent of RNA sequence was used.
(XLSX)

Table S2 **Summary of conserved and potato specific miRNA families.** The table shows the conserved miRNA families according to the family number corresponding to our analysis. The number of family members is indicated with mature miRNA and star sequences. The homologue of miRNA is shown (miRNA family) with the length of miRNA (miRNA length). For potato specific miRNAs the family number, numbers of miRNAs in the same family, mature and star sequences and miRNA length are indicated. During the analysis DNA equivalent of RNA sequence was used.
(XLSX)

Table S3 **Predicted target RNAs of conserved and potato specific miRNAs.** The table contains the miRNAs with the read number in the libraries (miRNA_Acc.), the homologue of miRNA (miRNA family) and the targeted potato transcripts (Target_Acc.) with the predicted gene functions (target gene function annotation). Expectation score (Expectation) and unpaired energy (UPE) with a low number suggest highly likely target for the miRNA. The aligned miRNA and target start and end nucleotide is indicated with the corresponding sequences. The inhibition type (Inhibition) and the number of target sites (multiplicity) are shown. On the second sheet of the table predicted targets could be found using more relaxed prediction criteria. During the analysis DNA equivalent of RNA sequence was used.
(XLSX)

Table S4 **Predicted targets of miR172, miR482 and validated potato specific miRNAs.** Details of miR172, miR482 and the validated miRNAs are shown in the table. The predicted target transcripts are below the miRNA data. For details see Table S1 and S3. During the analysis DNA equivalent of RNA sequence was used.
(XLSX)

Table S5 **Oligonucleotide sequences used in this study.**
(XLSX)
